# A Novel *SCN5A* Mutation in a Patient with Coexistence of Brugada Syndrome Traits and Ischaemic Heart Disease

**DOI:** 10.1155/2009/963645

**Published:** 2009-10-13

**Authors:** Anders G. Holst, Kirstine Calloe, Thomas Jespersen, Pernille Cedergreen, Bo G. Winkel, Henrik Kjaerulf Jensen, Trond P. Leren, Stig Haunso, Jesper Hastrup Svendsen, Jacob Tfelt-Hansen

**Affiliations:** ^1^The Danish National Research Foundation Centre for Cardiac Arrhythmia (DARC), University of Copenhagen, 2200 Copenhagen, Denmark; ^2^Laboratory of Molecular Cardiology, Department of Cardiology, Copenhagen University Hospital, Rigshospitalet, 2100 Copenhagen, Denmark; ^3^Department of Biomedical Sciences, University of Copenhagen, 2100 Copenhagen, Denmark; ^4^Department of Cardiology, Aarhus University Hospital Skejby, 8200 Aarhus, Denmark; ^5^Medical Genetics Laboratory, Rikshospitalet University Hospital, 0027 Oslo, Norway

## Abstract

Brugada syndrome (BrS) is a primary electrical heart disease, which can lead to sudden cardiac death. In older patients with BrS, the disease may coexist with ischaemic heart disease (IHD) and recent studies support a synergistic proarrhythmic effect of the two disease entities. We report a case that illustrates this. The index patient was a middle-aged patient with BrS traits, IHD, and aborted sudden cardiac death. Mutation analysis discovered a novel mutation P468L in the Na_V_1.5 sodium channel. Surprisingly, voltage-clamp experiments on the wild-type and mutant Na_V_1.5 channels expressed in HEK cells revealed no functional effect of the mutation. In a patient like ours, the distinction between IHD and BrS as the cause of an aborted sudden cardiac death is hard to establish and mounting evidence shows that coexistence of the two may have a synergistic proarrhythmic effect.

## 1. Introduction

The Brugada syndrome (BrS) is an inherited primary electrical heart disease. It is characterized by characteristic ST segment elevations in the right precordial leads of the ECG in patients with structurally normal hearts and a high frequency of malignant ventricular arrhythmias [1]. Patients with BrS are predominantly male, and the onset of symptoms is usually around the age of 40 years. The disease is believed to be rather common in South East Asia [2], but is rare in western countries [3]. Arrhythmic events are most common at rest or during sleep [4]. In 15–20% of BrS patients, the syndrome is associated with mutations in the *SCN5A* gene encoding the cardiac Na_V_1.5 sodium channel [5–7]. The inheritance is autosomal dominant with varying penetrance. In middle aged and elderly patients the phenotype of BrS and ischaemic heart disease (IHD) may coexists creating a mixed phenotype and hence possible diagnostic difficulties. Furthermore studies support a synergistic effect between BrS and IHD in the development of arrhythmic events [8, 9]. Here we describe a patient with BrS traits, IHD, and a novel missense mutation in the *SCN5A* gene with an apparently normal function.

## 2. Methods

### 2.1. Molecular-Genetic Analysis

DNA sequencing of the translated exons with flanking intron sequences of five genes encoding *KCNQ1*, *KCNH2*, *KCNE1*, *KCNE2*, and *SCN5A* was performed [10]. 

### 2.2. Functional and Biochemical Analysis of Na_V_1.5 Channels

HEK293 cells were transiently cotransfected with 0.3 *μ*g WT or P468L Na_V_1.5 constructs and 0.2 *μ*g of pcDNA3-EGFP. Human Na_V_1.5 in pcDNA3 was a gift from Dr. H. Abriel (Lausanne University).

## 3. Case Presentation

The patient was a 61-year-old Caucasian male with a history of angina pectoris and four prior elective coronary angiographies (CAG) in 1996, 1998, 2002, and 2003; all of these showing atheromatosis but no significant stenosis. He had a long history of cigarette smoking and hypertension.

**Figure 1:**
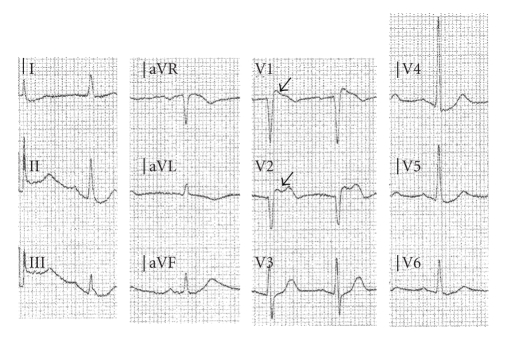
ECG (paper speed 25 mm/s) taken just after the patient was resuscitated. Notice the type-1 BrS configuration in V_1_ (upper arrow) and the type 2 configuration in V_2_ (lower arrow). All recordings were taken from the same ECG.

In December 2006, the patient experienced a syncope followed by chest pain and was transported to the nearest emergency department. Upon arrival he developed ventricular fibrillation, but after two direct current counter shocks sinus rhythm was restored. An electrocardiogram (ECG) taken immediately after resuscitation showed (Figure 1(a)) ST segment elevations in lead V_1_ and V_2_ with a type-1 BrS configuration in V_1_. Shortly, hereafter the patient was transferred from the local community hospital to a regional hospital to undergo an acute CAG. The CAG demonstrated a 90% stenosis on the circumflex coronary artery, which was treated with Percutaneous Coronary Intervention (PCI). TIMI flow both before and after stenting were 3. Blood samples taken at time of admission showed normal biochemistry, especially normal troponin T and creatinine kinases. Transthoracic echocardiography revealed borderline hypertrophy of the left ventricle but was otherwise normal.

Subsequently the patient underwent an invasive electrophysiological study (EPS) during which ventricular fibrillation was induced. Because of the EPS findings and clinical signs of BrS in conjunction with an episode of ventricular fibrillation, the patient received an implantable cardioverter-defibrillator (ICD). In the two years of follow up, no ICD therapies, appropriate or inappropriate, had been delivered (follow up until January 2009).

Years later a flecainide provocation test as well as an ECG with elevated electrode placement were performed, but none of these resulted in induction of a type-1 BrS ECG configuration, illustrating the variable nature of the ECG changes. A retrospective review of the patients ECGs though, uncovered an ECG from July 2006 showing a BrS Type-1 pattern in V_1_.

Furthermore, during the unraveling of the disease history it was revealed that many years ago, the patient had experienced two syncopes. 

### 3.1. Genetic Analysis

The genetic analysis revealed that the patient was heterozygous for a novel missense mutation P468L (c.1403C > T) in exon 11 of the *SCN5A* gene. The mutation was not identified in a group of 630 unrelated subjects referred for genetic testing. The patient did not have the H558R polymorphism.

### 3.2. Family History, Clinical Evaluation, and Genetic Testing of Family Members

**Figure 2:**
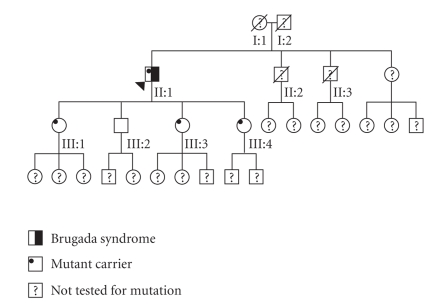
The family pedigree: the proband (II:1) is marked with an arrow.

The father (I:2) died at age 62 from lung cancer (Figure 2). The mother (I:1) died suddenly at age 64 but no cause of death was established. One brother (II:2) died at the age of 28, he had a heart valve operation in the years before his death, but no further information was available. Another brother (II:3) died at age 59. He had a history of ischaemic heart disease, but again no cause of death was known.

The patient had four children, three daughters and one son, in the age 30–42 years. All daughters carried the mutation, but not the son. None have had any known cardiac diseases or symptoms. The clinical examination revealed an incomplete right bundle branch block bearing resemblance to a Type 3 BrS configuration in the 42 year old daughter (III:4) ECG and a flecainide challenge was carried out but came out negative. ECGs from the last three children were normal.

### 3.3. Electrophysiological Characterization of Na_V_1.5 P468L

The peak current density did not reveal any difference between wild type and P468L mutant channels. To study the onset of fast inactivation, individual current traces were fitted using a biexponential function and the resulting time constants (*τ*) were plotted as a function of voltage. No significant differences in the *τ*_fast_ or *τ*_slow_ values of mutant channel compared to wild type were found. To further investigate the phenotype of Na_V_1.5 P468L, steady-state activation and inactivation were analyzed, as well as the voltage- and time-dependent release from inactivation (Table 1). Again, there was no difference between wild type and P468L channels.

## 4. Discussion

Cardiac enzymes after the cardiac arrest were normal, hence acute myocardial infarction (AMI) may be excluded, but transient ischemia cannot. The patients never exhibited diagnostic ECG changes for BrS, as a type-1 configuration was only seen in one lead and not the required two. In the clinical setting the patient was treated as having BrS though. The patient experienced two syncopes years prior to the cardiac arrest. The cause of the syncopes could be vasovagal, but self limiting episodes of ventricular tachycardia cannot be ruled out.

Genetic screening revealed a novel mutation in *SCN5A*. The mutation was found in 3 out of 4 of the progeny, but none had a BrS phenotype. The mutation not being present in 630 controls speaks in favor of the P468L substitution being the underlying cause of the BrS traits in the patient, but the lack of genotype/phenotype cosegregation in the family weakens the argument. The electrophysiological investigations of the mutation did not reveal any functional effect of the P468L substitution, and the substitution could be an innocent bystander instead of disease causing. However the mutated sodium channels expressed in HEK-293 cells may behave differently from in vivo due to lack of sodium channel interacting proteins, different trafficking or existence of a different set of polymorphisms.

Coexistence of BrS traits and IHD in a patient with ASCD can pose a problem in the clinical setting since the latter represents a potentially reversible cause of heart arrest and the former does not. Future ischemic events can in many cases be prevented by appropriate interventions whereas with the diagnosis of BrS and an episode of ASCD an ICD implantation is mandated [5].

As to the pathogenesis of the ASCD in this patient, the distinction between IHD and BrS as the substrate of sudden cardiac death is muddled by a number of reports showing that BrS increases the risk for arrhythmias during ischemia [8, 9] although not all studies support this conclusion [11].

The suggested mechanism for this accentuation of the risk of arrhythmias is the difference in the ion current balance (I_Na_/I_to_/*I*_CaL_) in epicardial versus endocardial cells of especially the right ventricular outflow tract. A balance that, according to this theory, is disturbed in BrS as well as during ischemia and the two in combination may have a synergistic proarrhythmic effect [12]. Furthermore, evidence is mounting that polymorphisms in *SCN5A* exons as well as in promoter regions has an impact on SCD susceptibility, albeit not specifically in the setting of IHD, but one could speculate that ischemia as well as these genetic changes both reduce the “antifibrillatory reserve” in a synergistic manner [13].

Currently many BrS patients are middle aged [14], and consequently we will in the near future see a large cohort of BrS patients coming into an age where IHD becomes prevalent and thus be facing the problems reported in this study. Further research into the modulating effect of BrS on the risk of SCD in the setting of IHD is warranted, as this could potentially lead to changes in risk stratification for BrS patients with high risk of IHD, as well as a better understanding of the arrhythmia inducing mechanisms underlying both.

In conclusion, we reported a case of aborted sudden cardiac death in a patient with coexistence of Brugada Syndrome traits, ischaemic heart disease, and a novel *SCN5A* mutation. The distinction between IHD and BrS as the cause of an aborted sudden cardiac death is hard to delineate, and is muddled by mounting evidence showing that coexistence of the two may have a synergistic proarrhythmic effect.

## Figures and Tables

**Table 1:** Kinetic parameters of wild type and P468L current measured in transiently transfected HEK-293 cells.

	Wild type	P468L
Peak current (at −20 mV)	−199 ± 35 pA/pF (*n* = 11)	−213 ± 39 pA/pF (*n* = 12)
Steady-state activation, V_1/2_	−30.4 ± 0.6 mV (*n* = 14)	−31.8 ± 0.6 mV (*n* = 10)
slope, *k* value	6.2 ± 0.3 mV/e-fold	6.2 ± 0.5 mV/e-fold
Steady-state inactivation, V_1/2_	−82.0 ± 1.3 mV (*n* = 13)	−79.2 ± 1.2 mV (*n* = 9)
slope, *k* value	6.2 ± 0.2 mV/e-fold	5.9 ± 0.3 mV/e-fold
